# Relationship between Subjective Hearing Loss and Work-related and Somatic Issues in the Working-age Population: A Large-scale Internet-based Cross-sectional Study

**DOI:** 10.31662/jmaj.2024-0430

**Published:** 2025-06-13

**Authors:** Jun Suzuki, Yuta Kobayashi, Hiyori Takahashi, Hiroki Tozuka, Shunsuke Takai, Ryoukichi Ikeda, Takahiro Tabuchi, Yukio Katori

**Affiliations:** 1 Department of Otolaryngology, Head and Neck Surgery, Tohoku University Graduate School of Medicine, Sendai, Japan; 2 Department of Otolaryngology, Head and Neck Surgery, Iwate Medical University School of Medicine, Yahaba, Japan; 3 Division of Epidemiology, Department of Health Informatics and Public Health, Tohoku University Graduate School of Public Health, Sendai, Japan; 4 Department of Community Medicine of Hearing Loss, Tohoku University Graduate School of Medicine, Sendai, Japan

**Keywords:** hearing loss, working-age, presenteeism, somatic symptoms

## Abstract

**Introduction:**

Hearing loss is a major problem that negatively impacts human life worldwide. Although factors associated with hearing loss have been widely studied, the impact of hearing loss on social aspects such as work performance in the working-age population remains unclear. To investigate the social and somatic problems associated with hearing loss in working-age individuals, we analyzed data from the Japan COVID-19 and Society Internet Survey (JACSIS), including a questionnaire concerning social aspects, mental health, physical health, and subjective hearing loss.

**Methods:**

We used the JACSIS 2023 data set, which includes data from 33,000 participants. After excluding participants with inappropriate responses and including those aged 20-64 years, data from 20,691 participants were used for further analysis. We compared various characteristics of the hearing loss group (moderate-to-severe subjective hearing loss) with those of the control group (no subjective hearing loss).

**Results:**

A total of 13,745 participants (male: 6,461; female: 7,284) were included in the control group, and 313 participants (male: 150; female: 163) were included in the hearing loss group. Multivariate logistic regression analysis revealed that both male and female participants in the hearing loss group exhibited higher incidences of tinnitus, presenteeism, somatic symptoms, and dyslipidemia compared with the control group. Additionally, subjective hearing loss was one of the independent explanatory variables for presenteeism in the working-age population.

**Conclusions:**

Our findings indicate significant associations between moderate-to-severe subjective hearing loss and various work-related and somatic factors in a working-age population, and encourage future research to assess whether subjective hearing loss independently contributes to presenteeism in the working-age population.

## Introduction

Hearing loss is a common problem worldwide, and without proper support, it can negatively affect many aspects of a person’s life. It impairs communication, limits educational opportunities, and can lead to social isolation, depression, and dementia, thus impairing social well-being ^(1), (2), (3), (4)^. The impact of hearing loss, especially in older people, is well-documented, with recent reports highlighting it as the largest potentially modifiable risk factor for dementia, accounting for 7% in the latest data ^(1), (5)^. The Global Burden of Disease study identifies hearing loss as the third most common disability, affecting 1.57 billion people worldwide ^(3)^, with an estimated annual global economic cost exceeding 981 billion US dollars ^(4)^. While numerous factors associated with hearing loss have been investigated ^(6), (7)^, its full impact remains unclear.

In addition to health problems, hearing loss negatively affects work status; people with hearing loss show increased levels of stress at work and a decreased ability to influence their work environment ^(8)^. Although the relationship among childhood hearing loss, education, and later employment is well established ^(9), (10)^, the social impact of other hearing losses on working-age individuals remains unclear. To address this gap, we focused on the Japan COVID-19 and Society Internet Survey (JACSIS) (https://jacsis-study.jp/) ^(11)^, which includes questions concerning social aspects, mental health, physical health, and hearing impairment.

JACSIS is a nationwide, cross-sectional study that gathers data from the pooled panels of a Japanese internet research agency (Rakuten Insight, Inc., https://insight.rakuten.co.jp/) ^(11)^. The JACSIS included community-dwelling panelists aged 15-79 years who were selected and invited to participate on the basis of random sampling stratified by sex, age, and prefecture. Herein, we investigated the factors associated with subjective moderate-to-severe hearing loss in the working-age population, focusing on somatic and work problems, including presenteeism (i.e., working while sick), using data from JACSIS.

## Materials and Methods

### Participants and data collection

We used data from JACSIS 2023, collected between September 25, 2023 and November 17, 2023. The data were accessed for research purposes on November 21, 2023. Among the initial 33,000 participants, 4,519 were excluded because of discrepancies and artificial/unnatural responses on the basis of a predefined exclusion algorithm established by the entire JACSIS project ^(12)^. We selected participants from the working-age population (aged 20-64 years). Consequently, the data from 20,691 participants were used for further analyses. Web-based informed consent was obtained from all participants upon registration. The research protocol of the JACSIS 2023 was approved by the Ethics Committee on Research of Human Subjects at the Osaka International Cancer Institute (number 20084). The revised version was approved by the Ethical Research Committee of the Tohoku University Graduate School of Medicine (number 2024-1-517).

### Definition of subjective hearing loss

In JACSIS 2023, we asked two questions about hearing loss: (1) “Throughout the last week, how much have you been bothered by hearing loss?” (answers: “not bothered at all,” “slightly bothered,” “a little bothered,” “considerably bothered,” and “severely bothered”); and (2) “Has your hearing loss lasted more than three months?” (answers: “yes” and “no”). To analyze the characteristics of participants with some degree of hearing difficulty, we defined participants who were considerably or severely bothered by hearing loss in the last week and whose hearing loss had lasted more than three months as the hearing loss group (moderate-to-severe subjective hearing loss cases). Conversely, participants who reported being “not bothered at all” by hearing loss in the past week and whose hearing loss had lasted less than 3 months were classified as the control group (no subjective hearing loss cases).

### Measures for hearing loss

To assess characteristics of participants with subjective hearing loss, we extracted and used the following information as covariates: sex, age, body mass index (BMI), marital status, educational level, alcohol, smoking, work with noise risk (agriculture, forestry, fisheries, fishing, mining, construction, manufacturing), work type, homeworking, the 8-item Somatic Symptom Scale (SSS-8) ^(13), (14)^, somatic symptoms in the last three months (vertigo, sleep disorder, and tinnitus), medical history (hypertension, diabetes, and dyslipidemia), the Kessler Psychological Distress Scale (K6) ^(15)^, the abbreviated Lubben Social Network Scale (LSNS‐6) ^(16)^, the Work Functioning Impairment Scale (WFun) ^(17)^, and absenteeism (sick absence).

The SSS-8 is a brief, self-administered questionnaire on the burden of somatic symptoms associated with depression, anxiety, general health status, and healthcare use ^(13), (14)^. We used the linguistically validated Japanese version of the SSS-8 ^(18)^. The total score ranges from 0 to 32 points, with severity categorized as no to minimal (0-3), low (4-7), medium (8-11), high (12-15), and very high (16-32). We adopted a cutoff score of 12 for this study to perform an analysis focusing on participants exhibiting high levels of somatic symptoms.

The K6 is a psychological questionnaire for screening individuals with possible mental illness (6 items, total 0-24 points) ^(15)^. We used the Japanese version of the K6 ^(19)^. People with a K6 score of ≥5 are usually considered to exhibit signs of mental health issues ^(20)^; accordingly, we used a cutoff score of 5.

The LSNS‐6 is a widely used measure of social isolation, with higher scores indicating stronger social networks (6 items, total 0-30 points) ^(16)^. It correlates with mortality, hospitalization, health behaviors, depressive symptoms, and overall physical health. We used the Japanese version of the LSNS-6 ^(21)^. Scores <12 are assumed to indicate social isolation ^(16)^; accordingly, we adopted a cutoff score of 12.

The WFun is a questionnaire developed in Japan that measures the degree of work functioning impairment due to health problems and is used to evaluate presenteeism (7 items, total 7-35 points) ^(17)^. The WFun severity was categorized as follows: low (7-13), mild (14-20), high (21-27), and very high (28-35) ^(17), (22)^. Individuals with a WFun score of ≥21 are considered to have significant work functioning impairment ^(17)^; thus, we used a cutoff score of 21 in this study.

To evaluate absenteeism, participants were asked to report the number of days of sick absence (only full days) due to their own health concerns in the past 30 days.

### Measures for presenteeism

We also evaluated the characteristics of participants with presenteeism using the same covariates as those used for hearing loss, along with additional variables previously associated with presenteeism: job demands, job control, and happiness ^(23)^. The presenteeism group included participants with a WFun score ≥21, while the control group included those with a WFun score <21.

Job demand was estimated using four items that assessed work characteristics: high volume, time consumption, hard work, and attention demand, referring to a previous study ^(24)^. Job control was rated using three items evaluating work characteristics: working at one’s own pace, deciding how to do work, and having one’s opinions reflected in the workplace. Responses were made on a 4-point scale: “yes” (1), “probably yes” (2), “probably no” (3), and “no” (4). Lower total scores indicate greater job demand (total 4-16) and greater job control (total 3-12).

Happiness, as a positive emotional state, was assessed using a one-item question: “In general, do you usually think you are happy?” Participants were required to answer on an 11-point scale, with 0 indicating “not at all applicable” and 10 indicating “completely applicable.” Following a previous study, we defined a cutoff of eight for happiness, which corresponded to the upper tertile point of all responses ^(23)^.

### Statistical analysis

#### Analyses concerning hearing loss

Differences between the control and hearing loss groups in the univariate analyses were evaluated using the Wilcoxon rank-sum test or chi-square test. Subsequently, we performed multivariate logistic regression analysis to investigate the association between hearing loss and various factors. Hearing loss was treated as the objective variable, and the explanatory variables were selected on the basis of clinical significance and factors showing significant differences in univariate analysis.

#### Analyses concerning presenteeism

Differences between the control and presenteeism groups in the univariate analyses were evaluated using the Wilcoxon rank-sum test or chi-square test. Subsequently, we performed multivariate logistic regression analysis to investigate the association between presenteeism and various factors. Presenteeism was treated as the objective variable, and the explanatory variables were selected on the basis of the clinical significance and factors showing significant differences in the univariate analysis.

Sex differences in the progression of age-related hearing loss are widely recognized, and thus analyses were performed separately for male and female participants ^(7), (25)^. To prevent multicollinearity, we confirmed that the variance inflation factors of all items were <2. All statistical analyses were performed using R version 4.4.1 (R Foundation for Statistical Computing, Vienna, Austria), and a two-tailed *P* <0.05 was considered statistically significant.

## Results

### Characteristics of the participants and univariate analyses concerning hearing loss

**Figure 1.**
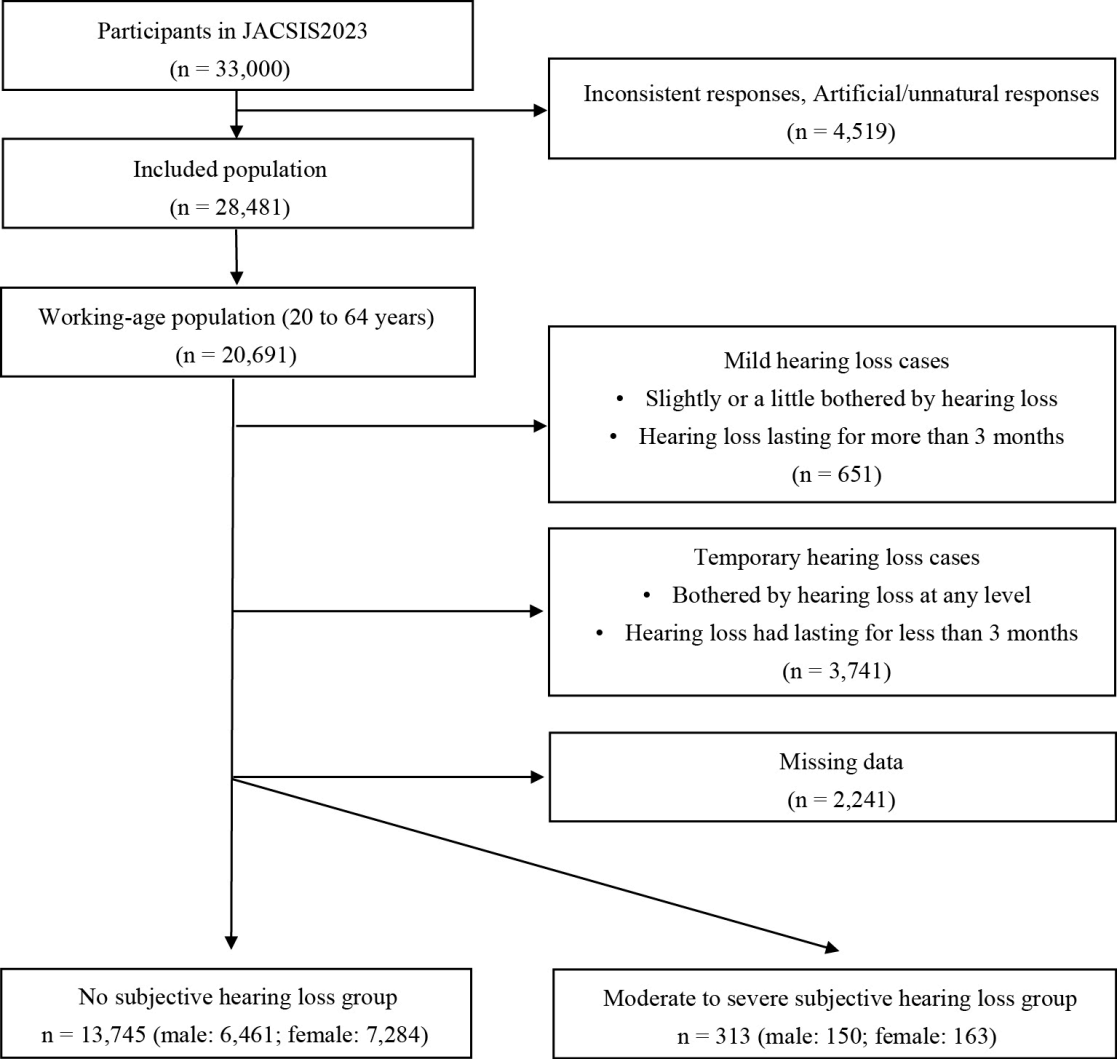
Study flow diagram for hearing loss analysis.

Of the 20,691 participants in the working-age population, 13,745 (male: 6,461; female: 7,284) were included in the control group, and 313 (male: 150; female: 163) were included in the hearing loss group (Figure 1).

Table 1 shows the characteristics of the male and female participants and the results of the univariate analyses. The characteristics of all participants who experienced subjective hearing loss for over three months are detailed in Supplementary Tables 1 and 2. Significant differences were observed between the male control and hearing loss groups in terms of age, BMI, marital status, smoking, SSS-8, vertigo, sleep disorders, tinnitus, hypertension, diabetes, dyslipidemia, K6, LSNS-6, WFun, and absenteeism.

**Table 1.** Characteristics of Participants with or without Subjective Hearing Loss.

Moderate to severe subjective hearing loss		Male			Female		
		No (n = 6461)	Yes (n = 150)	p-value	No (n = 7284)	Yes (n = 163)	p-Value
Age (years)		41.00 [29.00-52.00]	53.00 [37.00-60.00]	<0.001	40.00 [29.00-52.00]	50.00 [37.50-57.00]	<0.001
	20-34	2209 (34.2)	31 (20.7)	<0.001	2658 (36.5)	36 (22.1)	<0.001
	35-49	2209 (34.2)	34 (22.7)		2423 (33.3)	44 (27.0)	
	50-64	2043 (31.6)	85 (56.7)		2203 (30.2)	83 (50.9)	
BMI		22.46 [20.42-24.86]	23.32 [21.12-25.92]	0.015	20.20 [18.67-22.38]	21.23 [18.82-24.06]	0.003
Marital status	Married	3118 (48.3)	91 (60.7)	0.003	4018 (55.2)	84 (51.5)	0.4
	Single/divorce/bereavement	3343 (51.7)	59 (39.3)		3266 (44.8)	79 (48.5)	
Educational level	High school and below	1420 (22.1)	32 (21.6)	0.248	1674 (23.7)	60 (38.7)	<0.001
	Vocational school/college	831 (12.9)	26 (17.6)		2174 (30.7)	41 (26.5)	
	University and postgraduate	4175 (65.0)	90 (60.8)		3230 (45.6)	54 (34.8)	
Alcohol	No	590 (9.1)	11 (7.3)	0.262	1156 (15.9)	17 (10.4)	0.032
	Past	2143 (33.2)	59 (39.3)		3140 (43.1)	64 (39.3)	
	Current	3728 (57.7)	80 (53.3)		2988 (41.0)	82 (50.3)	
Smoking	No	2929 (45.3)	44 (29.3)	<0.001	5094 (69.9)	95 (58.3)	0.002
	Past	1878 (29.1)	60 (40.0)		1510 (20.7)	42 (25.8)	
	Current	1654 (25.6)	46 (30.7)		680 (9.3)	26 (16.0)	
Work with noise risk	No	4922 (76.2)	105 (70.0)	0.098	6561 (90.1)	147 (90.2)	1
	Yes	1539 (23.8)	45 (30.0)		723 (9.9)	16 (9.8)	
Work type	Desk work	2842 (48.9)	59 (44.4)	0.517	2497 (47.3)	58 (49.6)	0.216
	Sales work	1321 (22.7)	35 (26.3)		1446 (27.4)	24 (20.5)	
	Manual work	1653 (28.4)	39 (29.3)		1336 (25.3)	35 (29.9)
Somatic symptoms (SSS-8)		9.00 [6.00-12.00]	17.00 [12.00-21.00]	<0.001	10.00 [7.00-14.00]	17.00 [13.00-22.00]	<0.001
	No to medium	4183 (64.7)	29 (19.3)	<0.001	3954 (54.3)	20 (12.3)	<0.001
	High and more (≥12)	2278 (35.3)	121 (80.7)		3330 (45.7)	143 (87.7)	
Vertigo in 3 months	No	6225 (96.3)	117 (78.0)	<0.001	6735 (92.5)	102 (62.6)	<0.001
	Yes	236 (3.7)	33 (22.0)		549 (7.5)	61 (37.4)	
Sleep disorder in 3 months	No	5344 (82.7)	83 (55.3)	<0.001	5803 (79.7)	78 (47.9)	<0.001
	Yes	1117 (17.3)	67 (44.7)		1481 (20.3)	85 (52.1)	
Tinnitus in 3 months	No	6267 (97.0)	81 (54.0)	<0.001	7022 (96.4)	82 (50.3)	<0.001
	Yes	194 (3.0)	69 (46.0)		262 (3.6)	81 (49.7)	
Hypertension	No	4999 (77.4)	82 (54.7)	<0.001	6523 (89.6)	119 (73.0)	<0.001
	Past	392 (6.1)	12 (8.0)		252 (3.5)	11 (6.7)	
	Current	1070 (16.6)	56 (37.3)		509 (7.0)	33 (20.2)	
Diabetes	No	6047 (93.6)	127 (84.7)	<0.001	7079 (97.2)	144 (88.3)	<0.001
	Past	80 (1.2)	7 (4.7)		58 (0.8)	6 (3.7)	
	Current	334 (5.2)	16 (10.7)		147 (2.0)	13 (8.0)	
Dyslipidemia	No	5419 (83.9)	91 (60.7)	<0.001	6456 (88.6)	109 (66.9)	<0.001
	Past	279 (4.3)	9 (6.0)		201 (2.8)	12 (7.4)	
	Current	763 (11.8)	50 (33.3)		627 (8.6)	42 (25.8)	
Mental distress (K6 score)		2.00 [0.00-8.00]	8.50 [2.25-15.75]	<0.001	3.00 [0.00-8.00]	10.00 [3.50-17.00]	<0.001
	No	4024 (62.3)	51 (34.0)	<0.001	4349 (59.7)	45 (27.6)	<0.001
	Yes (≥5)	2437 (37.7)	99 (66.0)		2935 (40.3)	118 (72.4)	
Social isolation (LSNS-6)		9.00 [5.00-13.00]	6.50 [2.00-12.00]	<0.001	10.00 [6.00-14.00]	8.00 [4.00-12.00]	<0.001
	No	2303 (35.6)	39 (26.0)	0.019	3062 (42.0)	47 (28.8)	0.001
	Yes (<12)	4158 (64.4)	111 (74.0)		4222 (58.0)	116 (71.2)	
Presenteeism (WFun)		13.00 [8.00-20.00]	20.00 [13.00-27.00]	<0.001	12.00 [8.00-18.00]	19.00 [13.00-25.00]	<0.001
	No/mild	4501 (77.4)	70 (52.6)	<0.001	4338 (82.2)	65 (55.6)	<0.001
	Moderate/severe (≥21)	1315 (22.6)	63 (47.4)		941 (17.8)	52 (44.4)	
Absenteeism		0.00 [0.00-0.00]	0.00 [0.00-0.00]	<0.001	0.00 [0.00-0.00]	0.00 [0.00-0.00]	<0.001

Data are presented as median (interquartile range [IQR]). BMI: body mass index; K6: the Kessler Psychological Distress Scale; LSNS-6: the abbreviated Lubben Social Network Scale; SSS-8: 8-item Somatic Symptom Scale; WFun: Work Functioning Impairment Scale.

Significant differences were observed between the female control and hearing loss groups in terms of age, BMI, educational level, alcohol consumption, smoking, SSS-8, vertigo, sleep disorder, tinnitus, hypertension, diabetes, dyslipidemia, K6, LSNS-6, WFun, and absenteeism.

### Multivariate logistic regression analysis concerning hearing loss

We performed multivariate logistic regression analysis to identify factors independently associated with hearing loss. The distributions of the explanatory variables for the multivariate analysis and the results for male and female participants are presented in Table 2. The generalized variance inflation factors for each variable were <2 (Supplementary Table 3). Significant associations were observed for the following factors: the male hearing loss group demonstrated higher SSS-8 scores (odds ratio [OR]: 3.735), a higher incidence of tinnitus (OR: 17.519) and dyslipidemia (OR: 1.938 in the “current” group), higher K6 scores (OR: 1.715), and higher WFun scores (OR: 2.030) than the control group. Additionally, the female hearing loss group was older (OR: 2.454 in the “50-64” years group) and demonstrated a lower education level (OR: 0.556 in the “vocation/college” group), a higher percentage of current alcohol drinking (OR: 2.305), higher SSS-8 scores (OR: 3.088), a greater prevalence of vertigo (OR: 1.886), sleeping disorders (OR: 1.588), tinnitus (OR: 14.107), and dyslipidemia (OR: 1.780 in the “current” group), and higher WFun scores (OR: 2.161) than the control group.

**Table 2.** Multivariable Analysis for Subjective Hearing Loss.

	Male				Female			
	Odds ratio	95% CI (lower)	95% CI (upper)	p-Value	Odds ratio	95% CI (lower)	95% CI (upper)	p-Value
Age								
20-34	1 [reference]			-	1 [reference]			-
35-49	0.801	0.452	1.421	0.449	1.243	0.704	2.197	0.453
50-64	1.679	0.959	2.942	0.070	2.454	1.342	4.488	0.004
Education								
High school and below	1 [reference]			-	1 [reference]			-
Vocational school/college	1.460	0.793	2.690	0.225	0.556	0.323	0.955	0.033
University and postgraduate	0.914	0.563	1.485	0.717	0.702	0.418	1.180	0.182
Alcohol								
No	1 [reference]			-	1 [reference]			-
Past	1.247	0.540	2.878	0.606	1.252	0.567	2.767	0.579
Current	1.032	0.453	2.351	0.940	2.305	1.050	5.062	0.037
Smoking								
No	1 [reference]			-	1 [reference]			-
Past	1.320	0.801	2.176	0.275	1.191	0.734	1.934	0.479
Current	1.515	0.903	2.543	0.116	0.732	0.375	1.429	0.361
Somatic symptoms (SSS-8)								
No to medium	1 [reference]			-	1 [reference]			-
High and more (≥12)	3.735	2.245	6.214	<0.001	3.088	1.689	5.646	<0.001
Vertigo in 3 months								
No	1 [reference]			-	1 [reference]			-
Yes	1.474	0.836	2.602	0.180	1.886	1.139	3.121	0.014
Sleep disorder in 3 months								
No	1 [reference]			-	1 [reference]			-
Yes	1.437	0.948	2.178	0.087	1.588	1.021	2.470	0.040
Tinnitus in 3 months								
No	1 [reference]			-	1 [reference]			-
Yes	17.519	11.205	27.391	<0.001	14.107	8.786	22.652	<0.001
Hypertension								
No	1 [reference]			-	1 [reference]			-
Past	1.202	0.584	2.472	0.618	0.861	0.349	2.124	0.745
Current	1.512	0.951	2.404	0.081	1.680	0.918	3.077	0.093
Diabetes								
No	1 [reference]			-	1 [reference]			-
Past	2.256	0.804	6.328	0.122	2.904	0.749	11.262	0.123
Current	0.779	0.387	1.566	0.483	2.056	0.807	5.238	0.131
Dyslipidemia								
No	1 [reference]			-	1 [reference]			-
Past	1.007	0.427	2.378	0.986	2.532	1.132	5.661	0.024
Current	1.938	1.200	3.130	0.007	1.780	1.011	3.135	0.046
Mental distress (K6 score)								
No	1 [reference]			-	1 [reference]			-
Yes (5)	1.715	1.053	2.795	0.030	1.538	0.935	2.530	0.090
Social isolation (LSNS-6)								
No	1 [reference]			-	1 [reference]			-
Yes (<12)	1.034	0.670	1.594	0.881	0.883	0.560	1.391	0.590
Presenteeism (Fun)								
No/mild	1 [reference]			-	1 [reference]			-
Moderate/severe (≥21)	2.030	1.307	3.152	0.002	2.161	1.350	3.458	0.001

95% CI: 95% confidence interval; K6: Kessler Psychological Distress Scale; LSNS-6: the abbreviated Lubben Social Network Scale; SSS-8: 8-item Somatic Symptom Scale; WFun: Work Functioning Impairment Scale.

### Characteristics of the participants and univariate analyses concerning presenteeism

**Figure 2.**
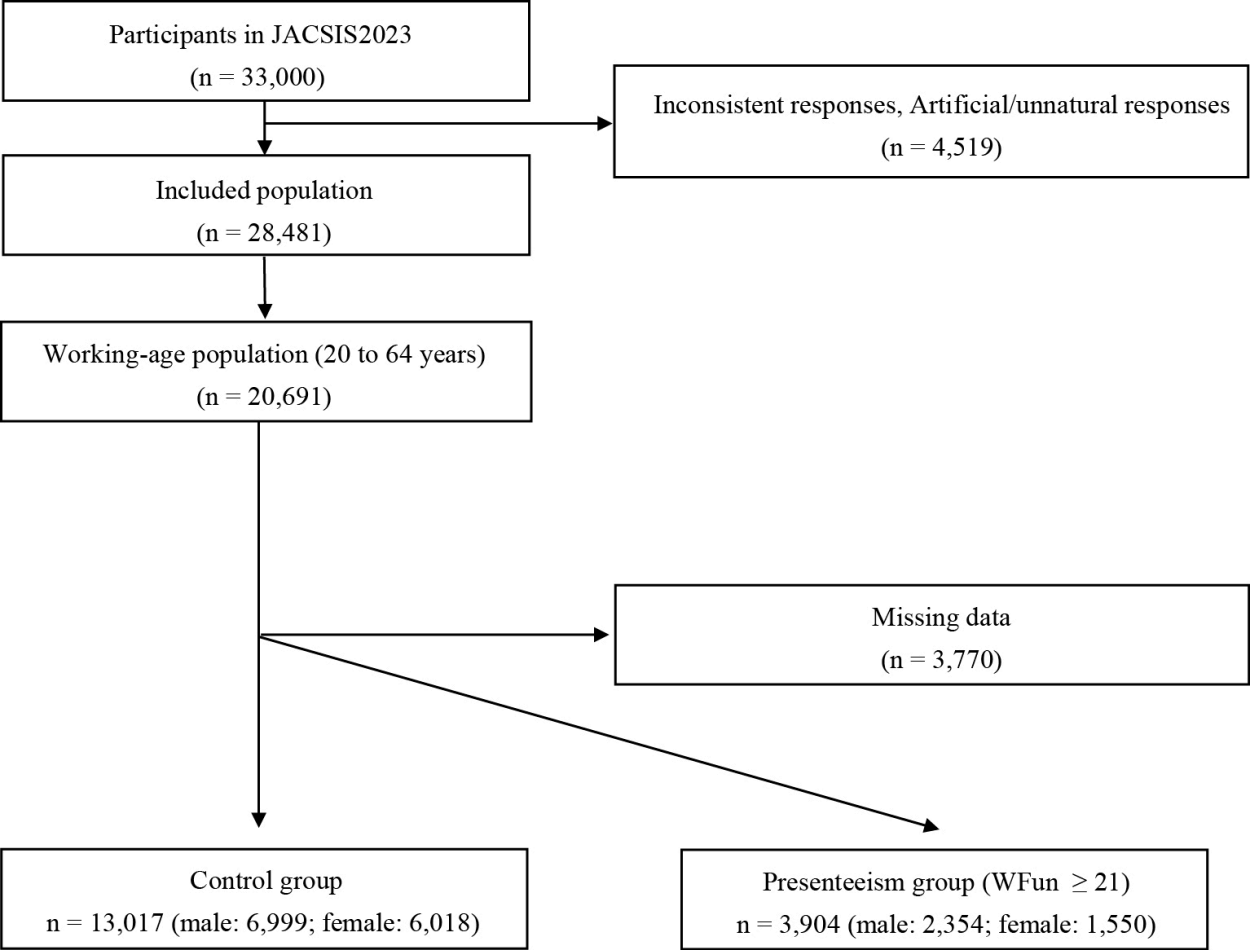
Study flow diagram for presenteeism analysis. WFun: Work Functioning Impairment Scale.

Since presenteeism was a significant explanatory variable for hearing loss in both male and female participants, we further assessed the characteristics of the participants, focusing on presenteeism. Of the 20,691 participants in the working-age population, 13,017 (male: 6,999; female: 6,018) were included in the control group and 3,904 (male: 2,354; female: 1,550) in the presenteeism group (Figure 2). Table 3 presents the characteristics of the male and female participants and the univariate analysis results. Significant differences between the presenteeism and control groups were observed across all explanatory variables except for smoking and tinnitus in male participants and BMI, alcohol, smoking, and working with noise in female participants.

**Table 3.** Characteristics of Participants with or without Presenteeism.

Presenteeism (Fun ≥21)		Male			Female		
		No (n = 6,999)	Yes (n = 2,354)	p-Value	No (n = 6,018)	Yes (n = 1,550)	p-Value
Subjective hearing loss (moderate to severe)	No	4,501 (98.5)	1,315 (95.4)	<0.001	4,338 (98.5)	941 (94.8)	<0.001
	Yes	70 (1.5)	63 (4.6)		65 (1.5)	52 (5.2)	
Age (years)		42.00 [30.00-53.00]	38.00 [29.00-48.00]	<0.001	41.00 [29.00-51.00]	35.00 [27.00-46.00]	<0.001
	20-34	2,257 (32.2)	945 (40.1)	<0.001	2,259 (37.5)	769 (49.6)	<0.001
	35-49	2,365 (33.8)	871 (37.0)		1,985 (33.0)	523 (33.7)	
	50-64	2,377 (34.0)	538 (22.9)		1,774 (29.5)	258 (16.6)	
BMI		22.49 [20.55-24.80]	22.31 [20.35-24.69]	0.043	20.13 [18.67-22.22]	20.07 [18.51-22.19]	0.129
Marital status	Married	3,761 (53.7)	1,142 (48.5)	<0.001	3,035 (50.4)	582 (37.5)	<0.001
	Single/divorce/bereavement	3,238 (46.3)	1,212 (51.5)		2,983 (49.6)	968 (62.5)	
Educational level	High school and below	1,562 (22.5)	454 (19.4)	0.003	1,426 (23.8)	303 (19.7)	<0.001
	Vocational school/college	933 (13.4)	304 (13.0)		1,787 (29.8)	414 (26.9)	
	University and postgraduate	4,450 (64.1)	1,586 (67.7)		2,781 (46.4)	821 (53.4)	
Alcohol	No	684 (9.8)	269 (11.4)	<0.001	991 (16.5)	262 (16.9)	0.313
	Past	2,185 (31.2)	902 (38.3)		2,484 (41.3)	666 (43.0)	
	Current	4,130 (59.0)	1,183 (50.3)		2,543 (42.3)	622 (40.1)	
Smoking	No	3,099 (44.3)	1,042 (44.3)	1	4,159 (69.1)	1,041 (67.2)	0.336
	Past	1,969 (28.1)	663 (28.2)		1,230 (20.4)	336 (21.7)	
	Current	1,931 (27.6)	649 (27.6)		629 (10.5)	173 (11.2)	
Work with noise risk	No	5,131 (73.3)	1,669 (70.9)	0.025	5,200 (86.4)	1,322 (85.3)	0.273
	Yes	1,868 (26.7)	685 (29.1)		818 (13.6)	228 (14.7)	
Work type	Desk work	3,328 (47.5)	1,258 (53.4)	<0.001	2,768 (46.0)	837 (54.0)	<0.001
	Sales work	1,597 (22.8)	523 (22.2)		1,690 (28.1)	352 (22.7)	
	Manual work	2,074 (29.6)	573 (24.3)		1,560 (25.9)	361 (23.3)	
Homeworking	No	5,240 (74.9)	1,583 (67.2)	<0.001	4,965 (82.5)	1,164 (75.1)	<0.001
	Sometimes	1,143 (16.3)	577 (24.5)		635 (10.6)	251 (16.2)	
	Frequently	616 (8.8)	194 (8.2)		418 (6.9)	135 (8.7)	
Job demand		9.00 [8.00-12.00]	8.00 [6.00-10.00]	<0.001	10.00 [8.00-12.00]	8.00 [6.00-10.00]	<0.001
Job control		7.00 [6.00-8.00]	7.00 [6.00-9.00]	<0.001	7.00 [6.00-9.00]	8.00 [6.00-9.00]	<0.001
Somatic symptoms (SSS-8)		8.00 [5.00-12.00]	13.00 [9.00-17.00]	<0.001	10.00 [6.00-14.00]	15.00 [10.00-19.00]	<0.001
	No to medium	4,771 (68.2)	818 (34.7)	<0.001	3,402 (56.5)	399 (25.7)	<0.001
	High and more (≥12)	2,228 (31.8)	1,536 (65.3)		2,616 (43.5)	1,151 (74.3)	
Vertigo in 3 months	No	5,601 (96.2)	2,032 (90.8)	<0.001	5,065 (92.3)	1,260 (83.8)	<0.001
	Yes	219 (3.8)	207 (9.2)		421 (7.7)	243 (16.2)	
Sleep disorder in 3 months	No	4,907 (84.3)	1,675 (74.8)	<0.001	4,412 (80.4)	1,054 (70.1)	<0.001
	Yes	913 (15.7)	564 (25.2)		1,074 (19.6)	449 (29.9)	
Tinnitus in 3 months	No	5,541 (95.2)	2,115 (94.5)	0.188	5,204 (94.9)	1,378 (91.7)	<0.001
	Yes	279 (4.8)	124 (5.5)		282 (5.1)	125 (8.3)	
Hypertension	No	5,418 (77.4)	1,680 (71.4)	<0.001	5,411 (89.9)	1,356 (87.5)	0.001
	Past	389 (5.6)	205 (8.7)		173 (2.9)	72 (4.6)	
	Current	1,192 (17.0)	469 (19.9)		434 (7.2)	122 (7.9)	
Diabetes	No	6,483 (92.6)	2,073 (88.1)	<0.001	5,829 (96.9)	1,464 (94.5)	<0.001
	Past	117 (1.7)	98 (4.2)		62 (1.0)	31 (2.0)	
	Current	399 (5.7)	183 (7.8)		127 (2.1)	55 (3.5)	
Dyslipidemia	No	5,848 (83.6)	1,847 (78.5)	<0.001	5,332 (88.6)	1,335 (86.1)	0.027
	Past	305 (4.4)	151 (6.4)		194 (3.2)	59 (3.8)	
	Current	846 (12.1)	356 (15.1)		492 (8.2)	156 (10.1)	
Happiness	No	4,838 (69.1)	2,064 (87.7)	<0.001	3,714 (61.7)	1,312 (84.6)	<0.001
	Yes (≥8)	2,161 (30.9)	290 (12.3)		2,304 (38.3)	238 (15.4)	
Mental distress (K6 score)		1.00 [0.00-5.00]	10.00 [4.00-14.00]	<0.001	2.00 [0.00-6.00]	10.00 [5.00-15.00]	<0.001
	No	5,049 (72.1)	627 (26.6)	<0.001	3,973 (66.0)	351 (22.6)	<0.001
	Yes (≥5)	1,950 (27.9)	1,727 (73.4)		2,045 (34.0)	1,199 (77.4)	
Social isolation (LSNS-6)		9.00 [5.00-14.00]	8.00 [4.00-13.00]	<0.001	11.00 [7.00-15.00]	8.00 [4.00-12.00]	<0.001
	No	2,813 (40.2)	747 (31.7)	<0.001	2,832 (47.1)	478 (30.8)	<0.001
	Yes (<12)	4,186 (59.8)	1,607 (68.3)		3,186 (52.9)	1,072 (69.2)	

Data are presented as median (interquartile range [IQR]). BMI: body mass index; K6: the Kessler Psychological Distress Scale; LSNS-6: the abbreviated Lubben Social Network Scale; SSS-8: 8-item Somatic Symptom Scale; WFun: Work Functioning Impairment Scale.

### Multivariate logistic regression analysis concerning presenteeism

Finally, we performed a multivariate logistic regression analysis to confirm whether hearing loss was independently associated with presenteeism. The distributions of explanatory variables for the multivariate analysis and the results for male and female participants are presented in Table 4. The generalized variance inflation factors for each variable were <2 (Supplementary Table 4). Significant associations were observed for the following factors: the male presenteeism group exhibited a higher percentage of hearing loss (OR: 1.719), younger age (OR: 0.713 in the “50-64” years group), a higher education level (OR: 1.230 in the “university and postgraduate” group), a lower percentage of current alcohol drinking (OR: 0.725), higher job demand (OR: 0.871), lower job control (OR: 1.056), higher SSS-8 scores (OR: 1.517), a lower percentage of individuals reporting happiness (OR: 0.899), and higher K6 scores (OR: 3.915) than the control group. Additionally, the female presenteeism group demonstrated a higher percentage of hearing loss (OR: 2.145), younger age (OR: 0.391 in the “50-64” years group), a higher education level (OR: 1.217 in the “university and postgraduate” group), higher job demand (OR: 0.876), higher SSS-8 scores (OR: 1.543), a lower percentage of individuals reporting happiness (OR: 0.854), higher K6 scores (OR: 3.085), and higher LSNS-6 scores (OR: 1.520) than the control group.

**Table 4.** Multivariable Analysis for Presenteeism.

	Male				Female			
	Odds ratio	95% CI (lower)	95% CI (upper)	p-Value	Odds ratio	95% CI (lower)	95% CI (upper)	p-Value
Subjective hearing loss (moderate to severe)
No	1 [reference]				1 [reference]			
Yes	1.719	1.115	2.651	0.014	2.145	1.354	3.396	0.001
Age								
20-34	1 [reference]			-	1 [reference]			-
35-49	0.895	0.761	1.053	0.183	0.727	0.607	0.870	<0.001
50-64	0.713	0.584	0.870	0.001	0.391	0.306	0.500	<0.001
Education								
High school and below	1 [reference]			-	1 [reference]			-
Vocational school/college	1.064	0.832	1.361	0.620	1.029	0.817	1.297	0.806
University and postgraduate	1.230	1.028	1.471	0.024	1.217	0.983	1.508	0.071
Alcohol								
No	1 [reference]			-	1 [reference]			-
Past	0.783	0.606	1.013	0.062	0.879	0.694	1.114	0.286
Current	0.725	0.564	0.932	0.012	0.998	0.786	1.268	0.989
Smoking								
No	1 [reference]			-	1 [reference]			-
Past	1.065	0.900	1.261	0.463	1.063	0.875	1.292	0.539
Current	0.923	0.772	1.103	0.380	0.839	0.638	1.104	0.209
Job demand	0.871	0.849	0.893	<0.001	0.876	0.852	0.901	<0.001
Job control	1.056	1.023	1.090	0.001	1.003	0.967	1.039	0.885
Somatic symptoms (SSS-8)						
No to medium	1 [reference]			-	1 [reference]			-
High and more (≥12)	1.517	1.306	1.763	<0.001	1.543	1.297	1.836	<0.001
Vertigo in 3 months								
No	1 [reference]			-	1 [reference]			-
Yes	1.060	0.770	1.459	0.719	1.196	0.922	1.551	0.177
Sleep disorder in 3 months							
No	1 [reference]			-	1 [reference]			-
Yes	1.047	0.877	1.250	0.611	1.146	0.950	1.382	0.156
Tinnitus in 3 months								
No	1 [reference]			-	1 [reference]			-
Yes	0.743	0.493	1.118	0.154	1.031	0.698	1.521	0.879
Hypertension								
No	1 [reference]			-	1 [reference]			-
Past	1.301	0.990	1.709	0.059	1.121	0.725	1.731	0.608
Current	0.925	0.751	1.138	0.459	0.948	0.663	1.355	0.769
Diabetes								
No	1 [reference]			-	1 [reference]			-
Past	1.043	0.581	1.873	0.888	1.226	0.566	2.657	0.606
Current	0.904	0.638	1.281	0.570	1.058	0.598	1.870	0.847
Dyslipidemia								
No	1 [reference]			-	1 [reference]			-
Past	1.248	0.902	1.726	0.181	1.293	0.845	1.978	0.236
Current	1.163	0.924	1.463	0.198	1.247	0.927	1.677	0.144
Happiness								
No	1 [reference]				1 [reference]			
Yes (≥8)	0.899	0.871	0.928	<0.001	0.854	0.824	0.885	<0.001
Mental distress (K6 score)								
No	1 [reference]			-	1 [reference]			-
Yes (≥5)	3.915	3.358	4.565	<0.001	3.085	2.580	3.690	<0.001
Social isolation (LSNS-6)								
No	1 [reference]			-	1 [reference]			-
Yes (<12)	1.143	0.982	1.330	0.084	1.520	1.281	1.803	<0.001

95% CI: 95% confidence interval; K6: Kessler Psychological Distress Scale; LSNS-6: the abbreviated Lubben Social Network Scale; SSS-8: 8-item Somatic Symptom Scale.

## Discussion

On the basis of a nationwide, large web-based cross-sectional study in Japan, we revealed several factors associated with subjective moderate-to-severe hearing loss in the working-age population. Both male and female participants in the hearing loss group reported higher levels of tinnitus, presenteeism, somatic symptoms, and dyslipidemia. Moreover, subjective moderate-to-severe hearing loss was an independent explanatory variable for presenteeism in the working-age population. These results provide new insights for future research on hearing loss at the working age and highlight the significance of addressing hearing loss to maintain the labor productivity of individuals.

Tinnitus is the perception of sounds in the ear or head when no corresponding external acoustic stimulus is present ^(26)^. A recent systematic review and meta-analysis estimated the prevalence of tinnitus in young, middle-aged, and older adults to be 10%, 14%, and 24%, respectively ^(27)^. Severe tinnitus is associated with hearing loss and impairs an individual’s quality of life ^(28)^. A previous review estimated the prevalence of severe tinnitus to be 2.3% ^(27)^. In this study, the prevalence of tinnitus lasting for three months was approximately 50% in the hearing loss group and 3% in the control group (Table 1). Considering the high OR of tinnitus (male: 17.5; female: 14.1) for subjective hearing loss, we hypothesized that chronic tinnitus significantly contributes to subjective hearing loss in moderate-to-severe cases. However, sound masking due to loud tinnitus may distort self-perceived hearing ability, leading to over- or underestimation of subjective hearing loss ^(29)^. Therefore, we acknowledge that the hearing loss group may have included participants with tinnitus without objective hearing loss. Our JACSIS data did not include detailed questionnaire items on hearing and tinnitus; therefore, a detailed examination of the relationship between tinnitus severity and subjective hearing loss is an issue for future research.

This study identified presenteeism, defined by WFun scores of ≥21, as an independent explanatory variable for subjective moderate-to-severe hearing loss. Conversely, subjective moderate-to-severe hearing loss was also found to independently explain presenteeism. Presenteeism is defined as going to work ill and has recently attracted attention as a significant factor affecting organizational performance, resulting in lost productivity ^(30)^. Physical and mental health issues contribute to presenteeism, with mental illnesses and musculoskeletal symptoms, such as neck pain, being the leading causes of presenteeism in Japan ^(31)^. Similarly, a large-scale study in the United States revealed that chronic back pain, mental illness, general anxiety, and severe migraines were the leading causes of daily productivity loss, with allergies and headaches incurring the highest annual costs ^(32)^. Since hearing loss has not been addressed as a health issue related to presenteeism in the studies mentioned above ^(30), (31), (32)^, the relationship between hearing loss and presenteeism has not attracted much attention to date. However, a large-scale survey of the Swedish working population demonstrated that hearing complaints and tinnitus are associated with occupational stressors, burnout symptoms, and performance-based self-esteem ^(33)^. Employees with hearing difficulties often show a lack of energy or experience fatigue ^(8)^, and reduced hearing ability in noisy environments is reported to be significantly associated with lower self-reported productivity ^(34)^. Given that 7% of workers in the United States without occupational noise exposure report hearing difficulties, 5% have tinnitus, and 2% have both ^(35)^, it is clear that the relationship between hearing loss and presenteeism warrants further investigation. Our results provide evidence of the association of subjective hearing loss (i.e., awareness of listening difficulties) with presenteeism in the working-age population, and suggest the need for the establishment of appropriate countermeasures for this issue.

Somatization refers to the experience of physical symptoms that cannot be attributed to an identifiable medical condition ^(36)^. Typical somatic symptoms include fatigue, sleep disturbance, and chronic pain, such as back pain, headache, and joint pain; the SSS-8 used in this study adopts these complaints as somatic symptoms ^(14)^. In the hearing loss group in this study, >80% of the participants had high SSS-8 scores (≥12), indicating higher somatization. High SSS-8 scores were an explanatory factor for subjective hearing loss, even after considering confounding factors such as sleep disorders and mental distress. Somatization is observed across various medical specialties, and in otolaryngology, symptoms such as dizziness and tinnitus are often associated with somatization ^(37)^. In contrast, symptoms such as hearing loss and hoarseness are more frequently associated with objective findings due to well-established testing methods ^(37)^. However, somatization exists on a spectrum; certain types of hearing loss, such as functional hearing loss, may involve more pronounced somatization, while others may show it to a lesser extent. A previous study of approximately 2,500 German participants found that the prevalence of somatized deafness was 6.9%, with 0.6% exhibiting severe symptoms ^(36)^. Moreover, hearing status is negatively associated with mental problems such as higher distress, depression, somatization, and loneliness in young and middle-aged adults ^(38)^. Our results suggest that individuals with moderate-to-severe subjective hearing loss may experience somatization as a manifestation of underlying mental distress. Since early recognition and intervention with cognitive behavioral therapy can improve or even resolve somatic symptoms ^(39)^, clinicians should consider these factors when treating individuals with subjective hearing loss.

Dyslipidemia, including hyperlipidemia, is a risk factor for hearing loss, especially in noise-induced hearing loss ^(40)^ and sudden sensorineural hearing loss ^(41)^. The supposed mechanisms of inner ear microcirculation impairment caused by lipid abnormalities are as follows: (1) decreased blood flow caused by increased blood viscosity; (2) microvascular embolism caused by activation of the fibrinolytic system; (3) structural changes in the stria vascularis or hair cells; and (4) sustained capillary constriction due to inhibition of vasodilator factors ^(42)^. However, the relationship between age-related hearing loss and dyslipidemia remains controversial. Although several large-scale studies have negated the hazardous effects of dyslipidemia on hearing loss ^(43), (44)^, other studies have suggested that dyslipidemia may be significantly related to hearing loss ^(45), (46)^. This study showed no significant association between hypertension or diabetes and subjective hearing loss, but did identify a significant association with dyslipidemia. The detailed mechanism is unknown, and future large-scale studies that include objective measures of hearing and assessment of the severity of subjective hearing loss are desirable.

### Limitations

This study has several limitations. First, since the study design was cross-sectional, we could not establish causality in the observed associations. However, this study provides essential data for considering the risk and moderating factors of subjective hearing loss in future studies. Second, because this study did not include hearing assessments, such as pure-tone audiometry, the hearing loss group may have included normal hearing cases. In addition, some individuals with objective hearing loss may not have reported symptoms of subjective hearing loss because of factors related to their work and family environment. However, given the increasing attention on hearing impairments with normal audiograms (such as hidden hearing loss ^(47)^ and listening difficulties/auditory processing disorders ^(48)^), we believe that including questions about subjective hearing symptoms is crucial when assessing the impact of hearing loss on individuals and society. Third, it was impossible to distinguish between congenital and adult-onset hearing loss. Given the low prevalence of congenital hearing loss compared with adult-onset or age-related hearing loss among community-dwelling participants, we expect the impact of this limitation on our conclusions to be small. Fourth, detailed information on the main variables of this study, such as tinnitus, was lacking. Therefore, future studies with more detailed information on auditory symptoms are warranted to evaluate the cross-sectional relationships between subjective hearing loss and other factors. Finally, the generalizability of the findings in this study might be limited because of its web-based, cross-sectional design, which restricted participation to only individuals with both internet access and sufficient motivation to complete the survey. However, because we included only working-age participants supposedly familiar with internet use and compared the two groups regarding subjective hearing loss and presenteeism, we believe that we could evaluate these items without significant bias.

### Conclusions

This is the first study to use large-scale internet data to identify the factors independently associated with subjective hearing loss in Japan. We identified several factors associated with moderate-to-severe subjective hearing loss in a working-age population, including presenteeism and somatic symptoms. Our findings encourage future investigations to assess whether subjective hearing loss independently contributes to presenteeism in the working-age population. Additionally, our findings motivate clinicians to consider integrating mental health screening and interventions, such as cognitive behavioral therapy, for individuals experiencing subjective hearing loss and somatic symptoms.

## Article Information

### Conflicts of Interest

None

### Sources of Funding

This study (JACSIS 2023) was supported by the Japan Society for the Promotion of Science KAKENHI Grants (grant number 21H04856; 23K18370), and the Health Labor Sciences Research Grants (grant number 22JA1005; 23EA1001; 23FA1004).

### Acknowledgement

This study (JACSIS 2023) was supported by the Japan Society for the Promotion of Science KAKENHI Grants (grant number 21H04856; 23K18370), and the Health Labor Sciences Research Grants (grant number 22JA1005; 23EA1001; 23FA1004). We thank Editage (www.editage.jp) for the English language editing.

### Author Contributions

Jun Suzuki, Yuta Kobayashi, and Takahiro Tabuchi designed the study.

Jun Suzuki, Yuta Kobayashi, and Takahiro Tabuchi performed data acquisition.

Jun Suzuki and Yuta Kobayashi analyzed and interpreted data.

Jun Suzuki, Yuta Kobayashi, Hiyori Takahashi, Hiroki Tozuka, Shunsuke Takai, Ryoukichi Ikeda, Takahiro Tabuchi, and Yukio Katori drafted the manuscript and revised it critically.

### Approval by Institutional Review Board (IRB)

The research protocol of JACSIS 2023 was approved by the Ethics Committee on Research of Human Subjects at the Osaka International Cancer Institute (no. 20084). The revised version was approved by the Ethical Research Committee of the Tohoku University Graduate School of Medicine (no. 2024-1-517).

### Data Availability Statement

Data are available upon reasonable request. Data from this study (JACSIS 2023) are not deposited in a public repository, as they contain personally identifiable or potentially sensitive information. Details of data availability can be found on the JACSIS website (https://jacsis-study.jp/dug/index.html).

## Supplement

Supplementary Tables
