# Supplementary material for: Relationship between Subjective Hearing Loss and Work-related and Somatic Issues in the Working-age Population: A Large-scale Internet-based Cross-sectional Study
Source: JMA J. 2025 Jun 13;8(3):753–65. doi: 10.31662/jmaj.2024-0430 (PMC12328264; doi:10.31662/jmaj.2024-0430)
Supplement: Supplementary Tables [file 2433-3298-8-3-0753-s001.pdf]

# Supplementary tables

## Supplementary Table 1

Characteristics of male participants with subjective hearing loss lasting for 3 months.

| Male | | No hearing loss | Slightly bothered | A little bothered | Considerably bothered | Severely bothered | |
|---|---|---|---|---|---|---|---|
| | | n=6461 | n=134 | n=167 | n=76 | n=74 | p-value |
| Age (years) | | 41.00 [29.00, 52.00] | 54.00 [39.00, 59.00] | 50.00 [34.00, 58.00] | 55.00 [41.00, 62.00] | 49.00 [36.25, 58.00] | <0.001 |
| | 20-34 | 2209 (34.2) | 25 (18.7) | 43 (25.7) | 14 (18.4) | 17 (23.0) | <0.001 |
| | 35-49 | 2209 (34.2) | 33 (24.6) | 39 (23.4) | 13 (17.1) | 21 (28.4) | |
| | 50-64 | 2043 (31.6) | 76 (56.7) | 85 (50.9) | 49 (64.5) | 36 (48.6) | |
| BMI | | 22.46 [20.42, 24.86] | 23.32 [21.34, 26.12] | 22.66 [20.76, 25.03] | 22.86 [21.13, 25.98] | 23.49 [21.12, 25.70] | <0.001 |
| Marital status | Married | 3118 (48.3) | 73 (54.5) | 92 (55.1) | 48 (63.2) | 43 (58.1) | 0.007 |
| | Single/divorce/bereavement | 3343 (51.7) | 61 (45.5) | 75 (44.9) | 28 (36.8) | 31 (41.9) | |
| Educational level | high school and below | 1420 (22.1) | 35 (26.7) | 50 (29.9) | 17 (22.4) | 15 (20.8) | 0.229 |
| | vocation/college | 831 (12.9) | 14 (10.7) | 20 (12.0) | 14 (18.4) | 12 (16.7) | |
| | university and postgraduate | 4175 (65.0) | 82 (62.6) | 97 (58.1) | 45 (9.2) | 45 (62.5) | |
| Alcohol | No | 590 (9.1) | 3 (2.2) | 10 (6.0) | 3 (3.9) | 8 (10.8) | 0.059 |
| | Past | 2143 (33.2) | 46 (34.3) | 58 (34.7) | 33 (43.4) | 26 (35.1) | |
| | Current | 3728 (57.7) | 85 (63.4) | 99 (59.3) | 40 (52.6) | 40 (54.1) | |
| Smoking | No | 2929 (45.3) | 34 (25.4) | 53 (31.7) | 20 (26.3) | 24 (32.4) | <0.001 |
| | Past | 1878 (29.1) | 39 (29.1) | 59 (35.3) | 35 (46.1) | 25 (33.8) | |
| | Current | 1654 (25.6) | 61 (45.5) | 55 (32.9) | 21 (27.6) | 25 (33.8) | |
| Work with noise risk | No | 4922 (76.2) | 107 (79.9) | 121 (72.5) | 51 (67.1) | 54 (73.0) | 0.197 |
| | Yes | 1539 (23.8) | 27 (20.1) | 46 (27.5) | 25 (32.9) | 20 (27.0) | |
| Work type | Desk work | 2842 (48.9) | 51 (45.5) | 76 (51.0) | 28 (41.2) | 31 (47.7) | 0.79 |
| | Sales work | 1321 (22.7) | 31 (27.7) | 37 (24.8) | 19 (27.9) | 16 (24.6) | |
| | Manual work | 1653 (28.4) | 30 (26.8) | 36 (24.2) | 21 (30.9) | 18 (27.7) | |
| Somatic symptoms (SSS-8) | | 9.00 [6.00, 12.00] | 11.00 [8.00, 15.00] | 13.00 [10.00, 17.00] | 15.00 [11.00, 19.25] | 18.00 [14.00, 21.75] | <0.001 |
| | No to medium | 4183 (64.7) | 66 (49.3) | 52 (31.1) | 17 (22.4) | 12 (16.2) | <0.001 |
| | High and more (>=12) | 2278 (35.3) | 68 (50.7) | 115 (68.9) | 59 (77.6) | 62 (83.8) | |
| Vertigo in 3 months | No | 6225 (96.3) | 114 (85.1) | 133 (79.6) | 61 (80.3) | 56 (75.7) | <0.001 |
| | Yes | 236 (3.7) | 20 (14.9) | 34 (20.4) | 15 (19.7) | 18 (24.3) | |
| Sleep disorder in 3 months | No | 5344 (82.7) | 84 (62.7) | 102 (61.1) | 43 (56.6) | 40 (54.1) | <0.001 |
| | Yes | 1117 (17.3) | 50 (37.3) | 65 (38.9) | 33 (43.4) | 34 (45.9) | |
| Tinnitus in 3 months | No | 6267 (97.0) | 99 (73.9) | 127 (76.0) | 40 (52.6) | 41 (55.4) | <0.001 |

| | | | | | | | |
|---|---|---|---|---|---|---|---|
| | Yes | 194 (3.0) | 35 (26.1) | 40 (24.0) | 36 (47.4) | 33 (44.6) | |
| Hypertension | No | 4999 (77.4) | 81 (60.4) | 99 (59.3) | 39 (51.3) | 43 (58.1) | <0.001 |
| | Past | 392 (6.1) | 9 (6.7) | 16 (9.6) | 7 (9.2) | 5 (6.8) | |
| | Current | 1070 (16.6) | 44 (32.8) | 52 (31.1) | 30 (39.5) | 26 (35.1) | |
| Diabetes | No | 6047 (93.6) | 110 (82.1) | 132 (79.0) | 63 (82.9) | 64 (86.5) | <0.001 |
| | Past | 80 (1.2) | 3 (2.2) | 11 (6.6) | 5 (6.6) | 2 (2.7) | |
| | Current | 334 (5.2) | 21 (15.7) | 24 (14.4) | 8 (10.5) | 8 (10.8) | |
| Dyslipidemia | No | 5419 (83.9) | 88 (65.7) | 115 (68.9) | 44 (57.9) | 47 (63.5) | <0.001 |
| | Past | 279 (4.3) | 11 (8.2) | 12 (7.2) | 6 (7.9) | 3 (4.1) | |
| | Current | 763 (11.8) | 35 (26.1) | 40 (24.0) | 26 (34.2) | 24 (32.4) | |
| Mental distress (K6 score) | | 2.00 [0.00, 8.00] | 3.50 [0.00, 10.00] | 6.00 [1.00, 12.00] | 7.50 [1.75, 13.00] | 11.50 [4.00, 20.00] | <0.001 |
| | No | 4024 (62.3) | 76 (56.7) | 69 (41.3) | 29 (38.2) | 22 (29.7) | <0.001 |
| | Yes (>=5) | 2437 (37.7) | 58 (43.3) | 98 (58.7) | 47 (61.8) | 52 (70.3) | |
| Social isolation (LSNS-6) | | 9.00 [5.00, 13.00] | 9.00 [4.00, 13.00] | 7.00 [4.00, 12.00] | 7.00 [4.75, 13.25] | 3.00 [1.00, 11.00] | <0.001 |
| | No | 2303 (35.6) | 50 (37.3) | 49 (29.3) | 22 (28.9) | 17 (23.0) | 0.051 |
| | Yes (<12) | 4158 (64.4) | 84 (62.7) | 118 (70.7) | 54 (71.1) | 57 (77.0) | |
| Presenteeism (Wfun) | | 13.00 [8.00, 20.00] | 15.00 [9.75, 21.00] | 18.00 [11.00, 24.00] | 19.00 [11.75, 25.25] | 20.00 [13.00, 28.00] | <0.001 |
| | No/mild | 4501 (77.4) | 79 (70.5) | 85 (57.0) | 37 (54.4) | 33 (50.8) | <0.001 |
| | Moderate/severe (>=21) | 1315 (22.6) | 33 (29.5) | 64 (43.0) | 31 (45.6) | 32 (49.2) | |
| Absenteeism | | 0.00 [0.00, 0.00] | 0.00 [0.00, 0.00] | 0.00 [0.00, 0.00] | 0.00 [0.00, 0.00] | 0.00 [0.00, 1.00] | <0.001 |

Data are presented as median (interquartile range). BMI, body mass index; SSS-8, 8-item Somatic Symptom Scale; K6, Kessler Psychological Distress Scale; LSNS-6, abbreviated Lubben Social Network Scale; WFun, Work Functioning Impairment Scale.

**Supplementary Table 2**

Characteristics of female participants with subjective hearing loss lasting for 3 months.

| Female | | No hearing loss | Slightly bothered | A little bothered | Considerably bothered | Severely bothered | |
|---|---|---|---|---|---|---|---|
| | | n=7284 | n=159 | n=191 | n=102 | n=61 | p-value |
| Age (years) | | 40.00 [29.00, 52.00] | 49.00 [39.00, 57.00] | 49.00 [38.50, 57.00] | 49.50 [38.00, 57.75] | 50.00 [37.00, 56.00] | <0.001 |
| | 20-34 | 2658 (36.5) | 28 (17.6) | 36 (18.8) | 21 (20.6) | 15 (24.6) | <0.001 |
| | 35-49 | 2423 (33.3) | 56 (35.2) | 67 (35.1) | 30 (29.4) | 14 (23.0) | |
| | 50-64 | 2203 (30.2) | 75 (47.2) | 88 (46.1) | 51 (50.0) | 32 (52.5) | |
| BMI | | 20.20 [18.67, 22.38] | 20.44 [19.01, 23.21] | 20.89 [18.82, 22.94] | 21.36 [19.18, 23.63] | 20.70 [18.20, 24.46] | 0.003 |
| Marital status | Married | 4018 (55.2) | 88 (55.3) | 109 (57.1) | 56 (54.9) | 28 (45.9) | 0.663 |
| | Single/divorce/bereavement | 3266 (44.8) | 71 (44.7) | 82 (42.9) | 46 (45.1) | 33 (54.1) | |
| Educational level | high school and below | 1730 (23.8) | 49 (30.8) | 51 (27.0) | 37 (36.6) | 24 (40.7) | <0.001 |
| | vocation/college | 2224 (30.6) | 53 (33.3) | 70 (37.0) | 25 (24.8) | 17 (28.8) | |
| | university and postgraduate | 3303 (45.5) | 57 (35.8) | 68 (36.0) | 39 (38.6) | 18 (30.5) | |
| Alcohol | No | 1156 (15.9) | 16 (10.1) | 22 (11.5) | 8 (7.8) | 9 (14.8) | 0.018 |
| | Past | 3140 (43.1) | 64 (40.3) | 88 (46.1) | 38 (37.3) | 26 (42.6) | |
| | Current | 2988 (41.0) | 79 (49.7) | 81 (42.4) | 56 (54.9) | 26 (42.6) | |
| Smoking | No | 5094 (69.9) | 92 (57.9) | 111 (58.1) | 54 (52.9) | 41 (67.2) | <0.001 |
| | Past | 1510 (20.7) | 49 (30.8) | 43 (22.5) | 31 (30.4) | 11 (18.0) | |
| | Current | 680 (9.3) | 18 (11.3) | 37 (19.4) | 17 (16.7) | 9 (14.8) | |
| Work with noise risk | No | 6561 (90.1) | 148 (93.1) | 175 (91.6) | 92 (90.2) | 55 (90.2) | 0.726 |
| | Yes | 723 (9.9) | 11 (6.9) | 16 (8.4) | 10 (9.8) | 6 (9.8) | |
| Work type | Desk work | 2497 (47.3) | 38 (35.8) | 55 (44.4) | 34 (46.6) | 24 (54.5) | 0.124 |
| | Sales work | 1446 (27.4) | 29 (27.4) | 36 (29.0) | 15 (20.5) | 9 (20.5) | |
| | Manual work | 1336 (25.3) | 39 (36.8) | 33 (26.6) | 24 (32.9) | 11 (25.0) | |
| Somatic symptoms (SSS-8) | | 10.00 [7.00, 14.00] | 12.00 [9.00, 16.50] | 15.00 [11.50, 19.00] | 17.00 [13.00, 22.00] | 18.00 [13.00, 24.00] | <0.001 |
| | No to medium | 3954 (54.3) | 61 (38.4) | 39 (20.4) | 10 (9.8) | 10 (16.4) | <0.001 |
| | High and more (>=12) | 3330 (45.7) | 98 (61.6) | 152 (79.6) | 92 (90.2) | 51 (83.6) | |
| Vertigo in 3 months | No | 6735 (92.5) | 120 (75.5) | 138 (72.3) | 66 (64.7) | 36 (59.0) | <0.001 |
| | Yes | 549 (7.5) | 39 (24.5) | 53 (27.7) | 36 (35.3) | 25 (41.0) | |
| Sleep disorder in 3 months | No | 5803 (79.7) | 81 (50.9) | 99 (51.8) | 47 (46.1) | 31 (50.8) | <0.001 |
| | Yes | 1481 (20.3) | 78 (49.1) | 92 (48.2) | 55 (53.9) | 30 (49.2) | |
| Tinnitus in 3 months | No | 7022 (96.4) | 117 (73.6) | 120 (62.8) | 50 (49.0) | 32 (52.5) | <0.001 |
| | Yes | 262 (3.6) | 42 (26.4) | 71 (37.2) | 52 (51.0) | 29 (47.5) | |
| Hypertension | No | 6523 (89.6) | 132 (83.0) | 147 (77.0) | 76 (74.5) | 43 (70.5) | <0.001 |

| | | | | | | | |
|---|---|---|---|---|---|---|---|
| | Past | 252 (3.5) | 8 (5.0) | 11 (5.8) | 8 (7.8) | 3 (4.9) | |
| | Current | 509 (7.0) | 19 (11.9) | 33 (17.3) | 18 (17.6) | 15 (24.6) | |
| Diabetes | No | 7079 (97.2) | 150 (94.3) | 177 (92.7) | 91 (89.2) | 53 (86.9) | <0.001 |
| | Past | 58 (0.8) | 4 (2.5) | 4 (2.1) | 3 (2.9) | 3 (4.9) | |
| | Current | 147 (2.0) | 5 (3.1) | 10 (5.2) | 8 (7.8) | 5 (8.2) | |
| Dyslipidemia | No | 6456 (88.6) | 125 (78.6) | 157 (82.2) | 66 (64.7) | 43 (70.5) | <0.001 |
| | Past | 201 (2.8) | 9 (5.7) | 10 (5.2) | 9 (8.8) | 3 (4.9) | |
| | Current | 627 (8.6) | 25 (15.7) | 24 (12.6) | 27 (26.5) | 15 (24.6) | |
| Mental distress (K6 score) | | 3.00 [0.00, 8.00] | 5.00 [1.50, 12.00] | 7.00 [3.00, 13.00] | 9.00 [3.00, 16.00] | 12.00 [5.00, 21.00] | <0.001 |
| | No | 4349 (59.7) | 74 (46.5) | 63 (33.0) | 30 (29.4) | 15 (24.6) | <0.001 |
| | Yes (>=5) | 2935 (40.3) | 85 (53.5) | 128 (67.0) | 72 (70.6) | 46 (75.4) | |
| Social isolation (LSNS-6) | | 10.00 [6.00, 14.00] | 9.00 [6.00, 13.00] | 9.00 [5.50, 13.50] | 8.00 [4.00, 12.00] | 7.00 [4.00, 13.00] | <0.001 |
| | No | 3062 (42.0) | 56 (35.2) | 66 (34.6) | 28 (27.5) | 19 (31.1) | 0.001 |
| | Yes (<12) | 4222 (58.0) | 103 (64.8) | 125 (65.4) | 74 (72.5) | 42 (68.9) | |
| Presenteeism (Wfun) | | 12.00 [8.00, 18.00] | 13.00 [9.00, 19.75] | 17.00 [11.00, 25.00] | 19.00 [14.00, 25.00] | 16.50 [11.00, 27.25] | <0.001 |
| | No/mild | 4338 (82.2) | 84 (79.2) | 79 (63.7) | 40 (54.8) | 25 (56.8) | <0.001 |
| | Moderate/severe (>=21) | 941 (17.8) | 22 (20.8) | 45 (36.3) | 33 (45.2) | 19 (43.2) | |
| Absenteeism | | 0.00 [0.00, 0.00] | 0.00 [0.00, 0.00] | 0.00 [0.00, 0.00] | 0.00 [0.00, 1.00] | 0.00 [0.00, 0.00] | <0.001 |

Data are presented as median (interquartile range). BMI, body mass index; SSS-8, 8-item Somatic Symptom Scale; K6, Kessler Psychological Distress Scale; LSNS-6, abbreviated Lubben Social Network Scale; WFun, Work Functioning Impairment Scale.

**Supplementary Table 3**

The generalized variance inflation factors for each variable in multivariate logistic regression analysis concerning hearing loss

| | Male | | | Female | | |
|---|---|---|---|---|---|---|
| | GVIF | Df | GVIF^(1/(2*Df)) | GVIF | Df | GVIF^(1/(2*Df)) |
| Age | 1.435431823 | 2 | 1.094575297 | 1.503585618 | 2 | 1.107342684 |
| Education | 1.082365313 | 2 | 1.019984252 | 1.218079247 | 2 | 1.050555222 |
| Alcohol | 1.117754448 | 2 | 1.028221313 | 1.123752597 | 2 | 1.029597969 |
| Smoking | 1.200137131 | 2 | 1.046665039 | 1.169496726 | 2 | 1.039919573 |
| Somatic symptoms (SSS-8) | 1.233508091 | 1 | 1.110634094 | 1.204272612 | 1 | 1.097393554 |
| Vertigo in 3 months | 1.115576067 | 1 | 1.056208345 | 1.187550722 | 1 | 1.089748009 |
| Sleep disorder in 3 months | 1.130663887 | 1 | 1.063326801 | 1.143934422 | 1 | 1.0695487 |
| Tinnitus in 3 months | 1.15543647 | 1 | 1.074912308 | 1.170220444 | 1 | 1.081767278 |
| Hypertension | 1.317041395 | 2 | 1.071272253 | 1.290491213 | 2 | 1.065832019 |
| Diabetes | 1.278840268 | 2 | 1.063418168 | 1.175729997 | 2 | 1.041302473 |
| Dyslipidemia | 1.34340855 | 2 | 1.076594178 | 1.273994524 | 2 | 1.062409365 |
| Mental distress (K6 score) | 1.503841408 | 1 | 1.226312117 | 1.342421233 | 1 | 1.158629032 |
| Social isolation (LSNS-6) | 1.053891908 | 1 | 1.026592377 | 1.104934426 | 1 | 1.051158611 |
| Presenteeism (Wfun) | 1.299161555 | 1 | 1.139807683 | 1.250031734 | 1 | 1.11804818 |

GVIF, generalized variance inflation factor; Df, degree of freedom; SSS-8, 8-item Somatic Symptom Scale; K6, the Kessler Psychological Distress Scale; LSNS-6, the abbreviated Lubben Social Network Scale; WFun, Work Functioning Impairment Scale.

**Supplementary Table 4**

The generalized variance inflation factors for each variable in multivariate logistic regression analysis concerning presenteeism

| | Male | | | Female | | |
|---|---|---|---|---|---|---|
| | GVIF | Df | GVIF^(1/(2*Df)) | GVIF | Df | GVIF^(1/(2*Df)) |
| Subjective hearing loss | 1.170643846 | 1 | 1.08196296 | 1.179347842 | 1 | 1.085977828 |
| Age | 1.333163743 | 2 | 1.074535761 | 1.335890172 | 2 | 1.075084718 |
| Education | 1.066155619 | 2 | 1.016143749 | 1.164261196 | 2 | 1.038753753 |
| Alcohol | 1.093237993 | 2 | 1.02253617 | 1.066975571 | 2 | 1.016339065 |
| Smoking | 1.173203522 | 2 | 1.040742619 | 1.12582839 | 2 | 1.030073107 |
| Job demand | 1.034079581 | 1 | 1.016897036 | 1.057785427 | 1 | 1.02848696 |
| Job control | 1.06300077 | 1 | 1.031019287 | 1.066380703 | 1 | 1.032657108 |
| Somatic symptoms (SSS-8) | 1.224064829 | 1 | 1.106374633 | 1.184881252 | 1 | 1.088522509 |
| Vertigo in 3 months | 1.083945018 | 1 | 1.041126802 | 1.109848822 | 1 | 1.053493627 |
| Sleep disorder in 3 months | 1.142920022 | 1 | 1.069074376 | 1.132250875 | 1 | 1.064072777 |
| Tinnitus in 3 months | 1.179217277 | 1 | 1.085917712 | 1.188775065 | 1 | 1.090309619 |
| Hypertension | 1.257604901 | 2 | 1.058975848 | 1.218740046 | 2 | 1.050697673 |
| Diabetes | 1.191073257 | 2 | 1.044683219 | 1.101975486 | 2 | 1.02457318 |
| Dyslipidemia | 1.261629708 | 2 | 1.059822113 | 1.187554942 | 2 | 1.043910889 |
| Happiness | 1.283275373 | 1 | 1.132817449 | 1.223716627 | 1 | 1.10621726 |
| Mental distress (K6 score) | 1.240155207 | 1 | 1.11362256 | 1.204828227 | 1 | 1.097646677 |
| Social isolation (LSNS-6) | 1.108138312 | 1 | 1.052681487 | 1.113482925 | 1 | 1.055217004 |

GVIF, generalized variance inflation factor; Df, degree of freedom; SSS-8, 8-item Somatic Symptom Scale; K6, the Kessler Psychological Distress Scale; LSNS-6, the abbreviated Lubben Social Network Scale.
